# GC × GC-TOFMS metabolomics analysis identifies elevated levels of plasma sugars and sugar alcohols in diabetic mellitus patients with kidney failure

**DOI:** 10.1016/j.jbc.2022.102445

**Published:** 2022-08-31

**Authors:** Kassaporn Duangkumpha, Narumol Jariyasopit, Kwanjeera Wanichthanarak, Esha Dhakal, Pattipong Wisanpitayakorn, Sansanee Thotsiri, Yongyut Sirivatanauksorn, Chagriya Kitiyakara, Nuankanya Sathirapongsasuti, Sakda Khoomrung

**Affiliations:** 1 Metabolomics and Systems Biology, Department of Biochemistry, Faculty of Medicine Siriraj Hospital, Mahidol University, Bangkok, Thailand; 2 Siriraj Metabolomics and Phenomics Center, Faculty of Medicine Siriraj Hospital, Mahidol University, Bangkok, Thailand; 3 Somdech Phra Debaratana Medical Center, Faculty of Medicine Ramathibodi Hospital, Mahidol University, Bangkok, Thailand; 4 Department of Medicine, Ramathibodi Hospital, Mahidol University, Bangkok, Thailand; 5 Research Network of NANOTEC - MU Ramathibodi on Nanomedicine, Bangkok, Thailand; 6 Section of Translational Medicine, Faculty of Medicine Ramathibodi Hospital, Mahidol University, Bangkok, Thailand; 7 Center of Excellence for Innovation in Chemistry (PERCH-CIC), Faculty of Science, Mahidol University, Bangkok, Thailand

**Keywords:** metabolomics, GC × GC-TOFMS, chronic kidney disease, kidney failure, diabetic mellitus, AUC, area under the curve, CKD, chronic kidney disease, CON, healthy controls, DKD, diabetic kidney disease, DM, diabetes mellitus, eGFR, estimated glomerular filtration rate, IS, internal standard, KF, kidney failure, OPLS-DA, orthogonal partial least squares discriminant analysis, SUS, shared and unique structure, TOFMS, time-of-flight mass spectrometry

## Abstract

Two dimensional GC (GC × GC)–time-of-flight mass spectrometry (TOFMS) has been used to improve accurate metabolite identification in the chemical industry, but this method has not been applied as readily in biomedical research. Here, we evaluated and validated the performance of high resolution GC × GC-TOFMS against that of GC-TOFMS for metabolomics analysis of two different plasma matrices, from healthy controls (CON) and diabetes mellitus (DM) patients with kidney failure (DM with KF). We found GC × GC-TOFMS outperformed traditional GC-TOFMS in terms of separation performance and metabolite coverage. Several metabolites from both the CON and DM with KF matrices, such as carbohydrates and carbohydrate-conjugate metabolites, were exclusively detected using GC × GC-TOFMS. Additionally, we applied this method to characterize significant metabolites in the DM with KF group, with focused analysis of four metabolite groups: sugars, sugar alcohols, amino acids, and free fatty acids. Our plasma metabolomics results revealed 35 significant metabolites (12 unique and 23 concentration-dependent metabolites) in the DM with KF group, as compared with those in the CON and DM groups (N = 20 for each group). Interestingly, we determined 17 of the 35 (14/17 verified with reference standards) significant metabolites identified from both the analyses were metabolites from the sugar and sugar alcohol groups, with significantly higher concentrations in the DM with KF group than in the CON and DM groups. Enrichment analysis of these 14 metabolites also revealed that alterations in galactose metabolism and the polyol pathway are related to DM with KF. Overall, our application of GC × GC-TOFMS identified key metabolites in complex plasma matrices.

Metabolomics analysis offers a great opportunity to study disease mechanisms, biomarkers, drug discovery, and precision medicine (1). Among all the metabolomics techniques, GC-MS has long been a standard technique for studying metabolites in various biological samples. This is because it offers the advantages of high sensitivity, robustness, excellent chromatographic separation, and availability of the libraries (2). In one-dimensional GC (1D GC), metabolites are solely separated on a column, on the basis of their boiling points and how they interact with the stationary phases (3). Although combining 1D GC with high resolution MS, such as time-of-flight mass spectrometry (TOFMS) or orbitrap-MS, can increase the separation power, the accuracy of metabolite identification remains an issue because of complex matrices and the separation performance (4). Clinical samples such as urine, blood, cells, or tissues are rich sources of metabolites but are highly complex and very difficult to analyze. To overcome this issue, comprehensive two-dimensional GC (GC × GC) has increasingly been applied to improve separation performance by using a series of two capillary GC columns with different stationary phases (5). High resolution GC × GC-TOFMS has recently been shown to serve as a superior method for increasing the confidence in metabolite identification of pesticides and a cannabidiol degradation products from cannabis samples (6). Although GC × GC has proven to be a promising technique for chemical characterization of a variety of samples over the last 30 years, its applications in the biomedical research have lagged behind those in other fields. From the current literature, the use of GC × GC in biomedical research has been relatively limited, whereas the majority of GC × GC applications have been in food and plant research, as well as petrochemical research (7).

Diabetes mellitus (DM) is a chronic disease characterized by high blood glucose levels due to ineffective insulin production or utilization (8). DM is among the top 10 major causes of mortality in adults, and its incidence is increasing globally (9). Diabetes is one of the leading causes of chronic kidney disease (CKD), characterized by elevated levels of albuminuria and decreased kidney function (as documented by elevated serum creatinine or decreased glomerular filtration rate) (10, 11). With time, patients with predialysis CKD develop a progressive decline in kidney function until they develop kidney failure (KF; also known as end-stage renal disease) and must require kidney replacement therapies by dialysis or transplantation to sustain life. In recent years, there has been an increasing interest in identifying new biomarkers for the prediction of CKD progression in predialysis diabetic kidney disease (DKD). Through metabolomics technology, various classes of candidate metabolites, including carbohydrates, amino acids, fatty acids, bile acids, and uremic solutes, have been reported to be linked to DM complications and kidney disease progression (12, 13, 14, 15). For instance, Niewczas *et al*. reported that the elevated levels of uremic toxins and polyols (sugar alcohols) are associated with the progression of CKD related to DM (15). Titan *et al*. reported that lactose 2-O-glycerol-α-galactopyranoside and tyrosine are significant metabolites related to KF progression (16).

To date, only a few studies have fully characterized the metabolomics profiles of DKD patients with established KF who are at very high risk of developing complications and mortality. Many earlier investigations used metabolomics to predict potential biomarkers, without using reference standards (12, 13, 14, 16). Many metabolites in patients with DM or DM with KF have very similar chemical structures and they are present at very low concentrations. Thus, determining the true identity and quantity of these metabolites in such a complicated matrix is highly challenging.

Despite the fact that GC × GC-TOFMS appears to have great potential for analyzing metabolites in clinical samples, its use in this area is currently limited, as compared to that of the standard 1D GC. Although GC × GC-TOFMS has been used to detect metabolites in blood samples in a few earlier studies, these studies were conducted on healthy individuals or samples from patients with early states of disease. Furthermore, it has long been known that GC × GC detects a greater number of features or metabolites than 1D GC, but the exact classes or groups of metabolites that are not detected in the 1D GC analysis are currently unknown. Therefore, the goal of this study was to establish and validate a GC × GC-TOFMS method using two different plasma matrices, for application in the identification of important metabolites in DM with KF patients.

## Results

### Implementation of sample preparation and GC × GC-TOFMS measurement conditions

We used the pooled plasma samples from the healthy control (CON) and DM with KF groups (N = 20 per group) to establish the method. The sample preparation protocol used in this study was adopted from a published protocol (17); however, we further optimized the key parameters that normally affect the silylation yields, that is, volume of supernatant (200 μl) and derivatizing agents (80 μl) as well as incubation time (∼25 min). For the GC × GC chromatographic separation, we used a nonpolar column as the first column, while a polar column was used as the second column. We found that an initial temperature of 50 °C was the best for concentrating the solvent and preventing the losses of small metabolites such as L-alanine, L-valine, and glycine. We also found that a 500 s acquisition delay (solvent delay) was the best setting for maximizing the detector lifetime and metabolite coverage.

### GC × GC-TOFMS extends the metabolites’ coverage over the traditional GC-TOFMS

**Figure 1:**
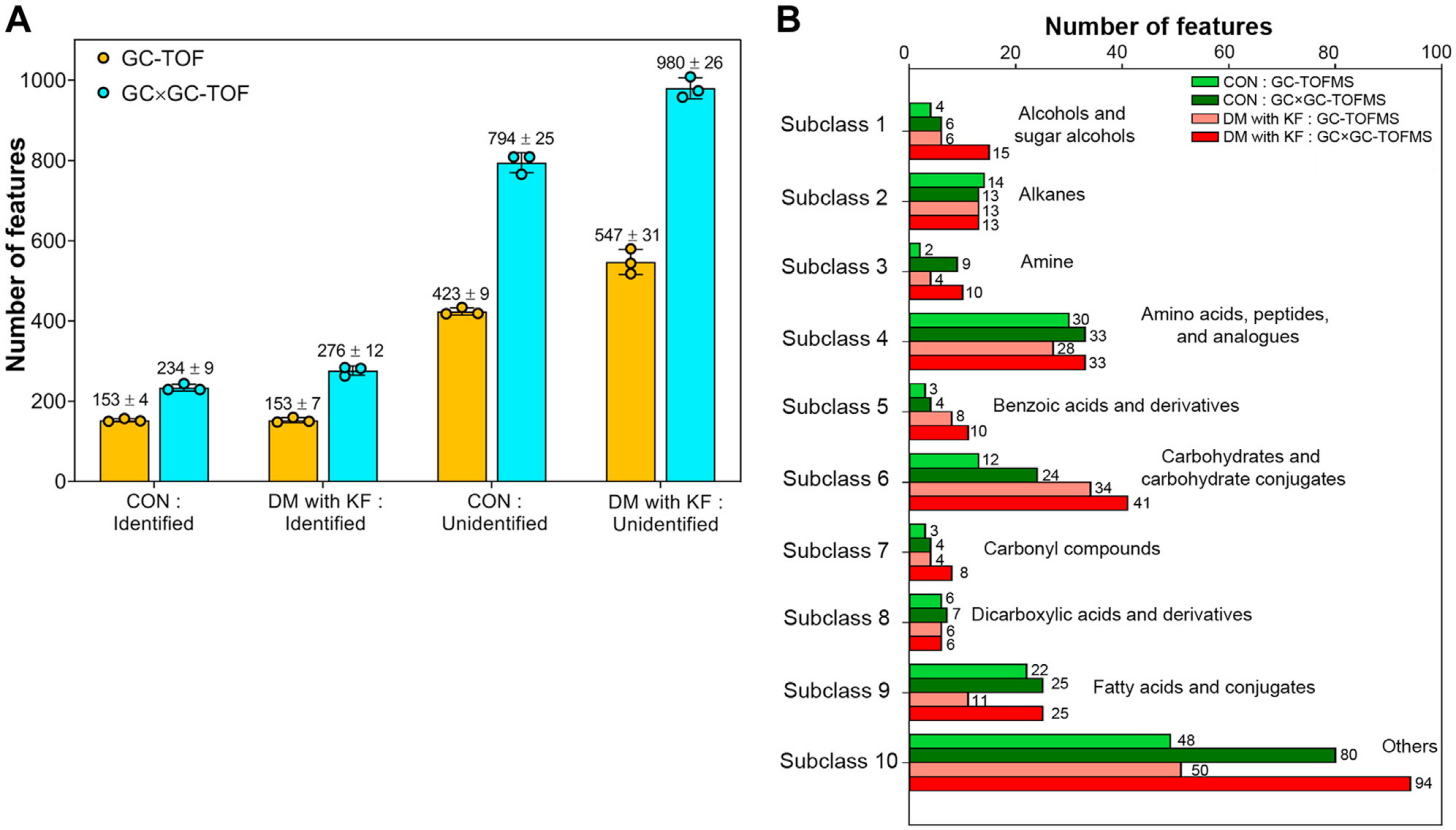
**Metabolites detected using GC-TOFMS and GC × GC-TOFMS in the pooled plasma samples from the CON (N = 3) and DM with KF (N = 3) groups.***A*, the scatter plot with bar charts shows the total number of features detected, including both identified and unidentified features. *B*, the identified features were classified into 10 subclasses based on the Human Metabolome Database. DM, diabetes mellitus; KF, kidney failure; TOFMS, time-of-flight mass spectrometry.

We evaluated the performance of GC × GC-TOFMS against traditional 1D GC (GC-TOFMS) using two separate pooled samples from the CON and DM with KF groups. The total number of features detected in the CON group was 576 ± 13 (153 ± 4 identified and 423 ± 9 unidentified) using GC-TOFMS and 1029 ± 16 (234 ± 9 identified and 794 ± 25 unidentified) using GC × GC-TOFMS (Fig. 1*A*). In the DM with KF group, the total number of features detected was 700 ± 26 (153 ± 7 identified and 547 ± 31 unidentified) using GC-TOFMS and 1257 ± 35 (276 ± 12 identified and 980 ± 26 unidentified) using GC × GC-TOFMS, respectively. In all cases, the number of features detected using GC × GC-TOFMS was clearly higher than detected using GC-TOFMS. Furthermore, we also observed a higher number of detected features (unannotated metabolite) in the DM with KF pooled samples, compared with that in the CON pooled samples. The most abundant of molecular sizes of the identified compounds in all matrices were in the range of 50 to 200 Da (Fig. S1). Based on the chemical taxonomy in the human metabolome database (18), the identified metabolites were categorized into 10 subclasses (Fig. 1*B*) (19). In subclasses such as subclass 1 (alcohols and sugar alcohols), subclass 3 (amines), subclass 6 (carbohydrates and carbohydrate conjugates), subclass 7 (carbonyl compounds), and subclass 9 (fatty acids and conjugates), the numbers of metabolites detected by GC × GC-TOFMS were at least two times higher than those detected by GC-TOFMS (Fig. 1*B*, Table S1). Other subclasses, including subclass 2 (alkanes), subclass 4 (amino acids, peptides, and analogs), subclass 5 (benzoic acids and derivatives), and subclass 8 (dicarboxylic acids and derivatives), showed slight or no differences between the numbers of metabolites detected by GC × GC-TOFMS and GC-TOFMS (Fig. 1*B*). Interestingly, many identified metabolites in subclass 6, such as arabinofuranose, D-arabinose, D-glucopyranose, galactopyranose, D-xylopyranose, and D-allofuranose, were exclusively detected using GC × GC-TOFMS in both the sample groups (Table S1). This was similar for the subclass 10 (others), which is a group of metabolites that could not be assigned to any class due to a small number of metabolites in the group (less than 5), and the availability of subclasses in the database (unclassified) Fig. S2*A* and S2*B*.

**Figure 2:**
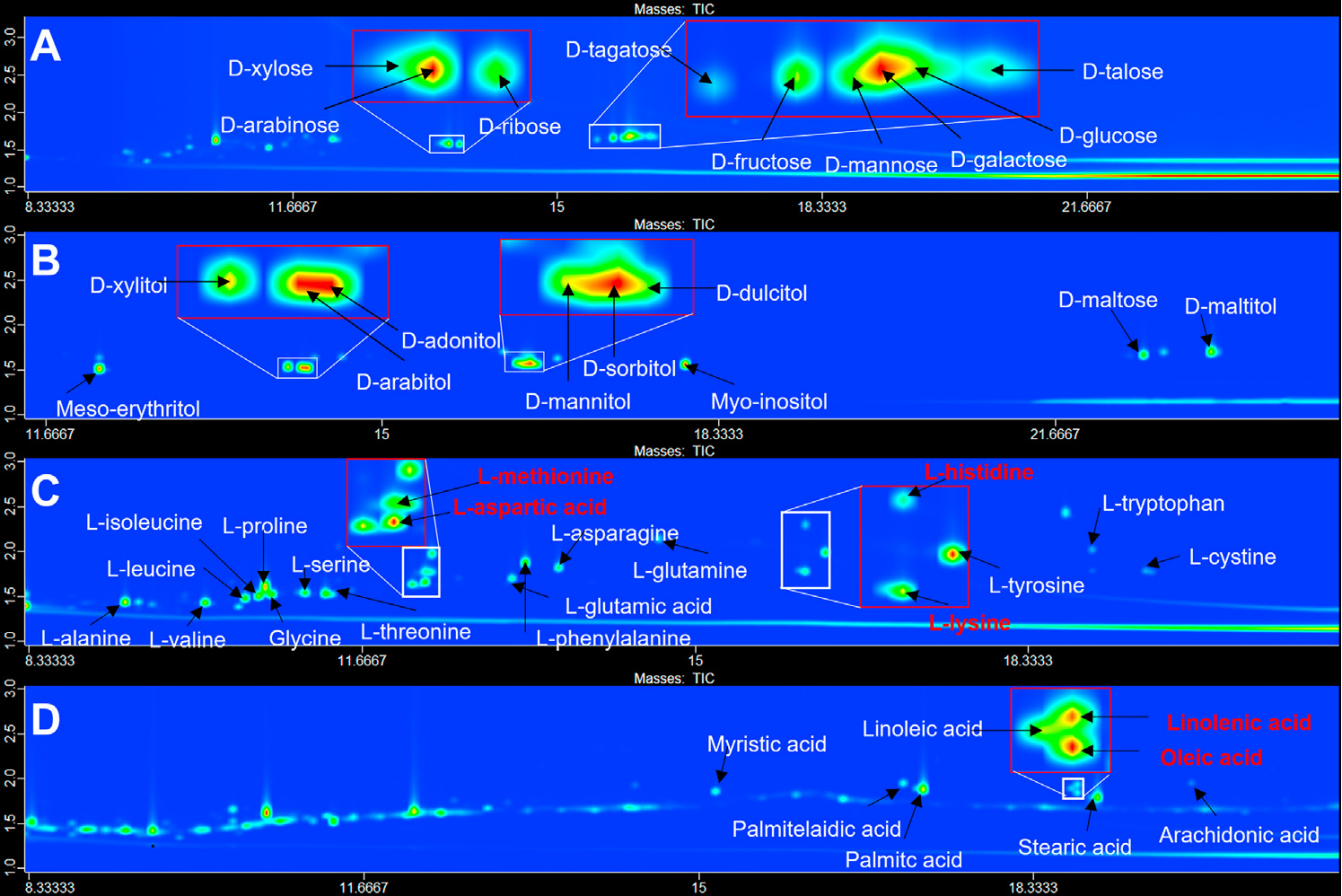
**GC × GC TOFMS demonstrated the four subclasses of reference standards.** Contour plots of the four subclasses of reference standards, including (*A*) ten sugar metabolites, (*B*) twenty amino acids, (*C*) eight fatty acids, and (*D*) nine sugar alcohols. The *x*- and *y*-axes represent the retention times in the first and second dimensions, respectively. The *red*-labeled metabolites indicate the coeluted compounds.

For validation of the targeted experiment, our metabolites of interest were selected based on previous reports (12, 13, 14, 15). We used 47 reference standards to set up the targeted GC × GC-TOFMS experiment. The standards included 9 sugar alcohols, 10 sugars, 20 amino acids, and 8 free fatty acids. With optimized chromatographic separation, over 98% (46/47) of the reference standards were clearly separated and correctly identified using our method (Fig. 2, *A*–*D*), with the exception of arginine, which was converted to ornithine during the trimethylsilyl derivatization (20). Although many derivative metabolites of amino acids (*i.e.*, aspartic acid, methionine, lysine, and histidine) and free fatty acids (*i.e.*, oleic acid and linolenic acids) were coeluted in the first column, they were well separated in the second dimension as shown in Fig. 2, *C* and *D*, respectively.

### Application of GC × GC-TOFMS to identify metabolites in DM with KF individuals characteristics of the study population

To search for significant metabolites in the DM with KF group, we recruited 60 participants from three groups: CON, DM, and DM with KF (N = 20 in each group). Table 1 summarizes the demographics, anthropometrics, and other clinical data of these individuals. Using the Kruskal–Wallis test, the key clinical parameters for CKD diagnosis, including estimated glomerular filtration rate (eGFR), creatinine, albumin, and total protein, were significantly different (*p* < 0.05) among the three groups, of which the DM with KF group exhibited abnormal values, as compared to those in the other groups (Table 1). The parameters of the kidney function test in the DM and CON groups were normal. The levels of fasting blood sugar levels in the DM and the DM with KF groups were significantly higher than those in the CON group.

**Table 1:** Baseline and clinical characteristics (mean ± SD) of the cohort

Characteristics	Reference range	CON (n=20)	DM (n=20)	DM with KF (n=20)	*p*-value
Age (year)		53 ± 7.03	56 ± 1.31	48 ± 8.72	
Gender, (male/female)		16/4	16/4	16/4	
Total cholesterol (mg/dl)^a^	<200	208 ± 32.91	194.40 ± 43.69	151.13 ± 0.60	0.004
AST (U/L)	5–34	22.58 ± 4.66	27.50 ± 9.98	25.40 ± 12.31	0.405
ALT (U/L)^a^	0–55	23.58 ± 7.5	36.60 ± 16.13	24.13 ± 20.25	0.009
Total bilirubin (mg/dl)	0.2–1.2	0.66 ± 0.31	0.67 ± 0.36	0.66 ± 0.13	0.618
Creatinine (mg/dl)^a^	Male = 0.73–1.18, Female = 0.55–1.02	0.94 ± 0.19	0.94 ± 0.19	9.45 ± 2.04	<0.001
eGFR (mL/min/1.73 m^2^)^a^	>90	90.09 ± 16.74	91.03 ± 11.59	5.66 ± 1.11	<0.001
Total protein (mg/dl)^a^	6.4–8.3	7.08 ± 0.68	7.43 ± 0.39	7.94 ± 0.83	<0.001
Albumin (g/L)^a^	3.5–5.0	4.49 ± 0.36	4.86 ± 0.27	3.95 ± 0.50	<0.001

AST, aspartate aminotransferase; ALT, alanine aminotransferase; eGFR, estimated glomerular filtration rate.

a *p* < 0.05, the Kruskal–Wallis test was used to compare the means among the three groups.

### Elevated levels of sugars and sugar alcohol metabolites in the DM with KF patients

**Figure 3:**
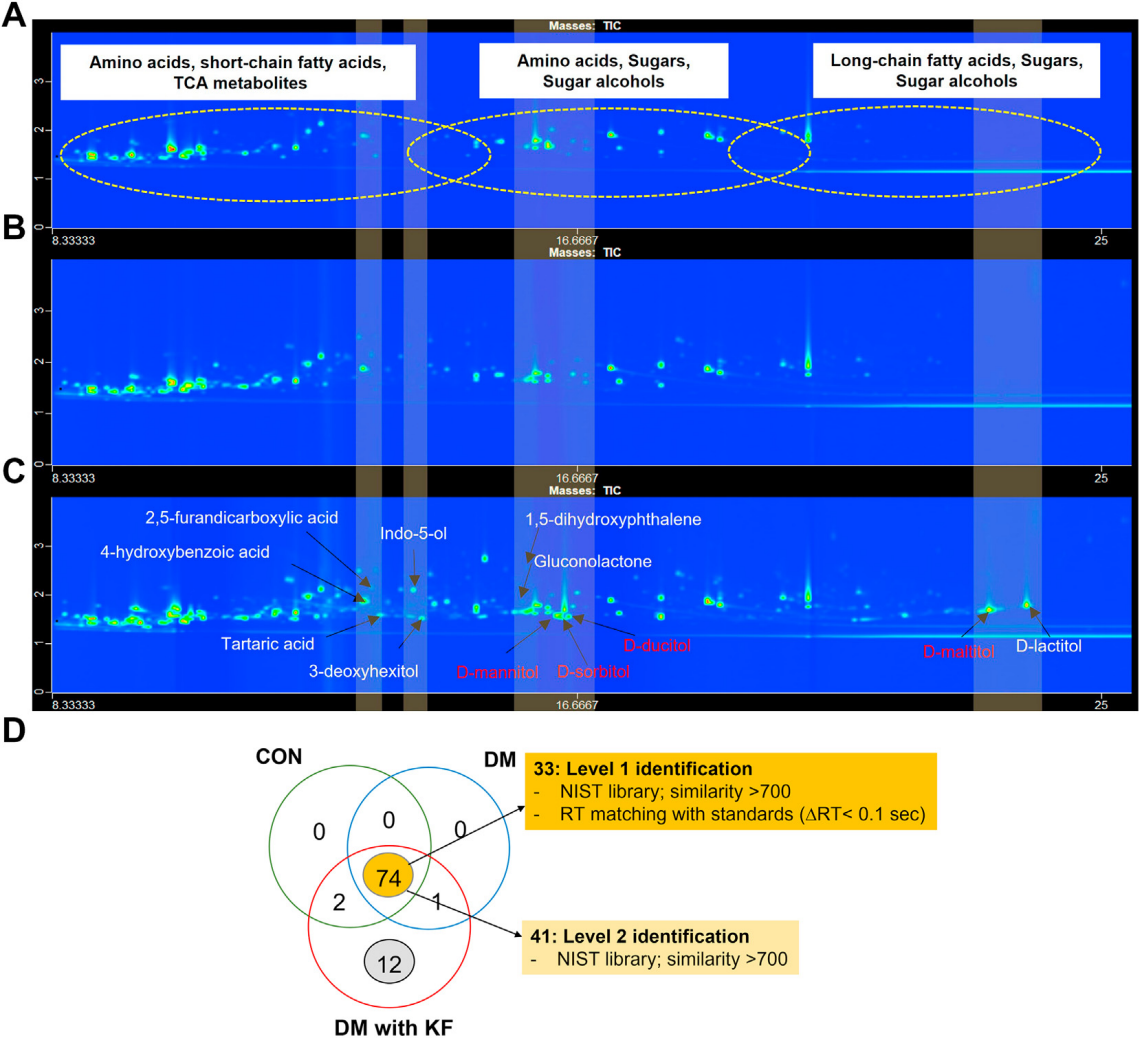
**Plasma metabolomic analysis in the cohort study using GC × GC TOFMS.** The contour plots of three samples representing the (*A*) CON, (*B*) DM, and (*C*) DM with KF groups; mannitol, sorbitol, dulcitol, and maltitol are the unique metabolites found in the DM with KF group (*red letters*). The *x*-axis represents the first dimension (min), while the *y*-axis represents the second dimension (sec). *D*, Venn diagram shows the number of identified metabolites in the three groups, including the CON, DM, and DM with KF groups. There were 74 common metabolites, which consisted of 33 and 41 metabolites, as identified based on level 1 and level 2 identifications, respectively. DM, diabetes mellitus; KF, kidney failure.

Overall, the metabolite profiles of the DM with KF group were clearly different from those of the CON and DM groups (Fig. 3, *A*–*C*), with sugar alcohols, oxidizing sugars, organic compounds, and other metabolites being exclusively present in the DM with KF samples (Fig. 3*C*). Based on the standard metabolite identification (21), we classified 89 metabolites (Table S2) into two metabolite identification levels. At the highest confidence level (level 1), detected features in the plasma samples were annotated with 33 reference standards, by comparing their MS spectra (>70% similarity matching) and retention times (<0.1 s for first and second dimension), with those of the standards analyzed using the identical measurement conditions. Level 2 identification was carried out by comparing the MS spectra of the unknown metabolites to those in the National Institute of Standard Technology (NIST) library (>70% similarity matching). Overall, there were 74 metabolites that were found to be common in all samples, while 12 metabolites were exclusively detected in the DM with KF group (Fig. 3*D*). We used two approaches to identify the key metabolites in the DM with KF group: (I) unique and (II) concentration-dependent metabolites. In the unique metabolite analysis, 4 of the 12 unique metabolites in the DM with KF group, namely, sugar alcohols, including D-mannitol, D-sorbitol, D-dulcitol, and D-maltitol, were verified and quantified using reference standards (Fig. S3). Their concentrations were 82.50 ± 29.42 μM for D-mannitol, 96.23 ± 69.39 μM for D-sorbitol, 23.89 ± 13.30 μM for D-dulcitol, and 259.63 ± 70.14 μM for D-maltitol. The remaining eight unique metabolites were annotated using the NIST library because of the limitations of the reference standards (Table 2).

**Table 2:** The significant candidate metabolites related to DM with KF

No.	Metabolite name	Metabolite class	Concentration in μM (mean ± SD)		
Unique metabolites in DM with KF					
1	D-maltitol^a^	sugar alcohol	259.63 ± 70.14		
2	D-sorbitol^a^	sugar alcohol	96.23 ± 69.39		
3	D-mannitol^a^	sugar alcohol	82.50 ± 29.42		
4	D-dulcitol^a^	sugar alcohol	23.89 ± 13.30		
5	D-lactitol	sugar alcohol	N/A		
6	Gluconolactone	oxidized sugar	N/A		
7	3-deoxyhexitol	reducing sugar	N/A		
8	Tartaric acid	organic compound	N/A		
9	4-hydroxybenzoic acid	organic compound	N/A		
10	2,5-furandicarboxylic acid	organic compound	N/A		
11	1,5-dihydroxyphthalene	other	N/A		
12	Indol-5-ol	other			

			Trend of metabolite level^b^		

CON *versus* DM	CON *versus* DM with KF	DM *versus* DM with KF

Concentration dependent metabolites					
1	D-arabinose^a^	sugar	↑	↑∗∗∗∗	↑∗∗∗∗
2	D-fructose^a^	sugar	↑	↑∗∗∗∗	↑∗∗∗∗
3	D-galactose^a^	sugar	↑	↑∗∗∗∗	↑∗∗∗∗
4	D-maltose^a^	sugar	↑	↑∗∗∗∗	↑∗∗∗∗
5	D-ribose^a^	sugar	↑	↑∗∗∗∗	↑∗∗∗∗
6	D-xylose^a^	sugar	↑	↑∗∗∗∗	↑∗∗∗∗
7	D-talose^a^	sugar	↑	↑∗∗∗∗	↑∗∗∗∗
8	Sucrose	sugar	↑	↑∗∗	↑∗∗∗∗
9	D-arabitol^a^	sugar alcohol	↑	↑∗∗∗∗	↑∗∗∗∗
10	Meso-erythritol^a^	sugar alcohol	↑	↑∗∗∗∗	↑∗∗∗∗
11	Myo-inositol^a^	sugar alcohol	↑	↑∗∗∗∗	↑∗∗∗∗
12	1,5-anhydroglucitol	sugar alcohol	↓	↓∗∗∗∗	↓∗∗∗∗
13	D-gluconic acid	oxidized sugar	↑	↑∗∗∗∗	↑∗∗∗∗
14	Methyl galactoside	oxidized sugar	↑	↑∗∗∗∗	↑∗∗∗∗
15	DL-Ornithine	amino acid	↑	↑∗∗∗∗	↑∗∗∗∗
16	Nonanoic acid	fatty acid	↑	↓∗∗∗∗	↓∗∗∗∗
17	Pseudouridine	nucleoside	↑	↑∗∗∗∗	↑∗∗∗∗
18	2,3,4-trihydroxybutyric acid	organic compound	↑	↑∗∗∗∗	↑∗∗∗∗
19	Benzoic acid	organic compound	↑	↓∗∗∗∗	↓∗∗∗∗
20	Hippuric acid	organic compound	↑	↑∗∗∗∗	↑∗∗∗∗
21	p-cresol	organic compound	↑	↑∗∗∗∗	↑∗∗∗∗
22	L-tryptophan^a^	amino acid	↓	↓∗∗∗∗	↓∗∗∗∗
23	L-tyrosine^a^	amino acid	↓	↓∗∗∗∗	↓∗∗∗∗

DM, diabetes mellitus; KF, kidney failure.

a Metabolites were verified with reference standards.

b *p*-values were calculated using the Mann–Whitney U test for pairwise comparisons (∗*p* < 0.05, ∗∗*p* < 0.01, ∗∗∗*p* < 0.001, and ∗∗∗∗*p* < 0.0001).

**Figure 4:**
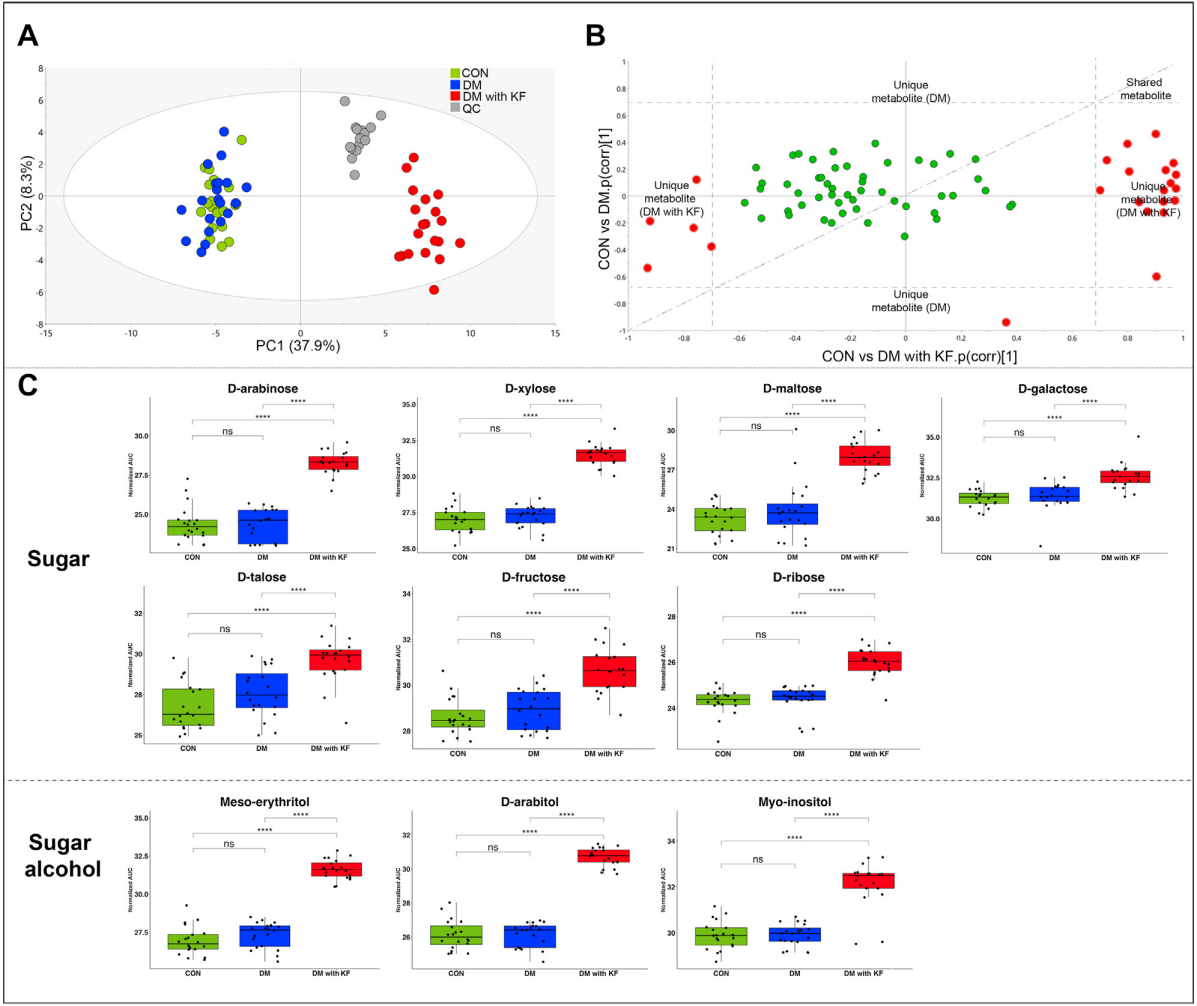
**Identification of significant metabolites in the DM with KF group.***A*, PCA score plot of the CON (*green*), DM (*blue*), DM with KF (*red*), and QC (*gray*) groups, (*B*) SUS plot of the 74 concentration-dependent metabolites related to DM with KF (*x*-axis) and DM (*y*-axis). *Red* circles represent the correlated metabolites with *p*(corr) > 0.7 (dashed line), in both directions of each condition. *C*, box plots represent the two main classes of significantly elevated metabolites in the DM with KF group, including seven sugar metabolites and three sugar alcohols. DM, diabetes mellitus; KF, kidney failure.

For concentration-dependent metabolite analysis (74 metabolites), we performed principal component analysis and observed a clustering of the pooled samples, which separated them from the other groups (Fig. 4*A*). This suggests that the metabolomics data were of high quality and had low analytical variance. There was a clear separation between the DM with KF group and the two other groups (Fig. 4*A*). However, no clear difference was observed between the DM and CON groups. By using a shared and unique structure (SUS) plot (22), we identified 23 significant metabolites [*p*(corr) > 0.7] in the DM with KF group, as compared to those in the CON and DM groups (Fig. 4*B*). Interestingly, 43% (10/23) of the significant metabolites (verified with the standards) in this analysis were from the sugar and sugar alcohol subclasses. These metabolites included D-xylose, D-arabinose, D-maltose, D-ribose, D-fructose, D-galactose, D-talose, meso-erythritol, D-arabitol, and myo-inositol, which were significantly elevated (*p* < 0.0001) in the DM with KF group, as compared to those in the CON and DM groups (Fig. 4*C*). The SUS plot also showed no clear difference between the CON and DM groups. A summary of the list and trends of the significant metabolites related to DM with KF is presented in Table 2.

### Alteration of carbohydrate metabolism in DM with KF patients

To determine the disturbed metabolic pathway in the DM patients with KF, we performed the enrichment analysis using seven sugar and three sugar alcohols that were identified in the concentration-dependent analysis, together with the four sugar alcohols identified in the unique metabolite analysis. We found that these 14 metabolites were associated with carbohydrate metabolism, including galactose metabolism, pentose and glucuronate interconversion, and fructose and mannose metabolism (Fig. S4). In particular, galactose metabolism was clearly impacted in the DM with KF group, exhibiting increased in levels of six sugar metabolites when compared to the CON and DM groups, namely D-galactose, sucrose, D-fructose, D-dulcitol, D-sorbitol, and myo-inositol in the DM with KF group (Table S3).

### Correlation between the 33 verified metabolites and clinical parameters

**Figure 5:**
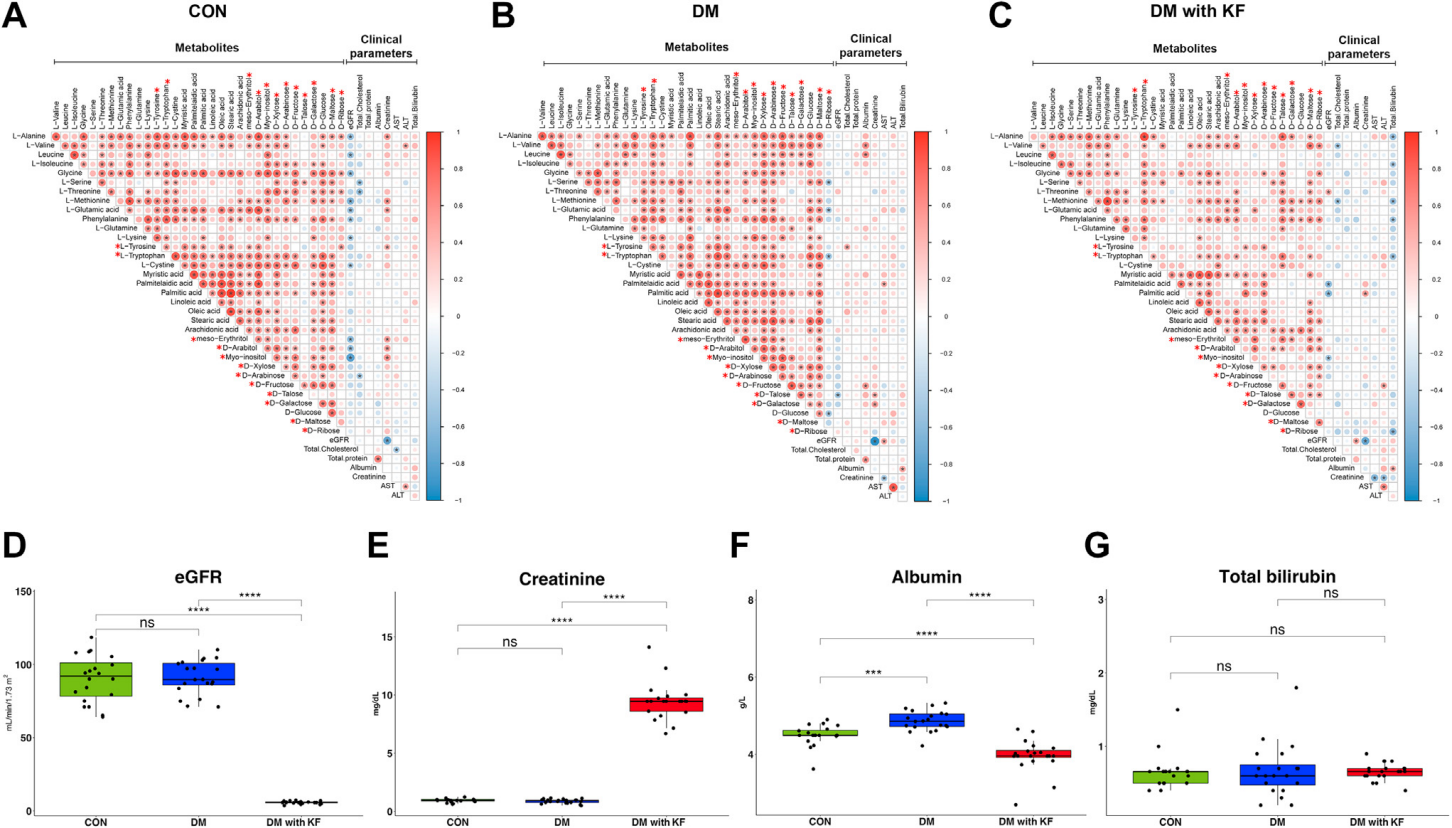
**Correlation analysis among the clinical parameters and verified metabolites.** The correlation coefficient represents the correlation between the 33 verified metabolites and clinical parameters in three sample groups, including CON (*A*), DM (*B*), and (*C*) DM with KF (*C*), as assessed using Spearman’s correlation analysis. Metabolites marked with *red* asterisks represent significant metabolites that were identified from the SUS plot analysis. The size and color intensity of the dots represent the size of the correlation coefficient values. The *red* and *blue* colors represent positive and negative correlation, respectively, while the blank indicates no correlation. Box plots of eGFR (*D*), creatinine (*E*), albumin (*F*), and total bilirubin (*G*) levels. ns (not significant) *p*-value > 0.05, ∗*p*-value < 0.05, ∗∗*p*-value < 0.01, ∗∗∗*p*-value < 0.001, and ∗∗∗∗*p*-value < 0.0001, as determined by the Mann-Whitney U test. DM, diabetes mellitus; eGFR, estimated glomerular filtration rate; KF, kidney failure; SUS, shared and unique structure.

We examined the correlation patterns between metabolite abundances and clinical parameters in each sample group (Fig. 5, *A*–*C*). Spearman’s rank correlation coefficients between the 33 verified metabolites and 10 clinical parameters, including age, sex, eGFR, total cholesterol, total protein, albumin, creatinine, aspartate aminotransferase, alanine aminotransferase, and total bilirubin, were computed. Clinical parameters with more than 50% missing values were excluded. The correlation was considered statistically significant, if its *p*-value was less than 0.05 (*p*< 0.05). A distinct correlation pattern among the 33 metabolites was observed in the DM with KF group. In particular, there were a smaller number of significant correlations in the DM with KF group than those in other groups. Only 190 positive correlations existed in the DM with KF group, whereas 300 and 289 significant pairs of correlated metabolites were found in the DM and CON groups, respectively. However, we found an increased correlation between D-talose and other metabolites in the DM with KF group. In this group, the D-talose specifically correlated with glycine, L-threonine, L-methionine, L-glutamic acid, phenylalanine, L-tryptophan, L-cystine, arachidonic acid, meso-erythritol, D-arabitol, D-maltose, and D-ribose. D-talose was positively correlated with L-valine, leucine, palmitic acid, stearic acid, myo-inositol, D-arabinose, and D-glucose in the DM group. There was a much less significant correlation in the CON samples.

Moreover, significant correlations between a group of metabolites and kidney function parameters, including eGFR, creatinine, albumin, and total bilirubin, were remarkably altered under the different disease conditions. In the CON group, the metabolites including L-alanine, leucine, glycine, L-methionine, L-glutamic acid, phenylalanine, L-lysine, L-tyrosine, L-cystine, meso-erythritol, D-arabitol, and myo-inositol showed strong positive and negative correlations with creatinine and eGFR, respectively. These associations were observed less frequently in patients with DM. Instead, explicit negative correlations between total bilirubin and L-alanine, L-isoleucine, L-methionine, phenylalanine, L-tryptophan, and D-ribose were observed in the DM with KF group. Additionally, we observed the absolute levels of these kidney function parameters and compared the differences in them among the groups. Both eGFR and serum creatinine levels were significantly lower in the DM with KF group than those in the other groups (Fig. 5, *D* and *E*). Albumin levels were significantly different, whereas total bilirubin levels were not statistically different among the groups (Fig. 5, *F* and *G*).

## Discussion

Our study showed that the performance of GC × GC-TOFMS was significantly better than that of traditional GC-TOFMS in terms of separation performance and metabolite coverage. The numbers of identified metabolites in the GC × GC-TOFMS analysis was almost twice of that measured using GC-TOFMS. The validated GC × GC-TOFMS method was found to be suitable for metabolite profiling and targeted analysis in a single run. To answer our research question, we targeted metabolite profiling of four classes of metabolites including sugars, sugar alcohols, amino acids, and free fatty acids. We demonstrated the improved separation of coeluted metabolites using the second column of the GC × GC-TOFMS instrument. This also increased the number of detected metabolites (identified metabolites based on reference standards or library) as well as the sensitivity of the metabolite profiling experiment. In addition to the targeted analytes, we were able to detect other metabolites from the groups of alcohols and sugar alcohols, amines, carbohydrates and carbohydrate conjugates, and fatty acids and conjugates. Interestingly, the optimized GC × GC-TOFMS method could detect several sugar forms; for example, both pyranose and furanose forms were only detected using the GC × GC-TOFMS analysis. The reducing sugar metabolites gave multiple peaks, due to the presence of anomers, pyranoside, and furanoside rings, and an open chain and cyclic form. This may result in extremely complex chromatographic peaks for the carbohydrate metabolites. Our findings demonstrated that GC × GC-TOFMS could detect the cyclic forms of sugars, such as D-glucose (open chain form), D-glucopyranose (cyclic form), D-galactose (openchain form), and D-galactopyranose (cyclic form), which cannot be resolved and identified using GC-TOFMS. Our findings indicated that GC × GC-TOFMS provides a higher separation power, particularly for the differentiation of sugar metabolites. Although several metabolites were uniquely detected using GC-TOFMS, these numbers were much lower than those detected using GC × GC-TOFMS (Table S2). Nevertheless, these results provided broad information about the metabolite coverage detected in plasma matrices using GC-TOFMS and GC × GC-TOFMS.

In addition, our GC × GC-TOFMS results are also consistent with a previous study by Winnike *et al*., who carried out human serum metabolomics and found that the GC × GC-TOFMS method provided better metabolite coverage, peak resolution, peak capacity, and sensitivity, as compared to those provided by GC-TOFMS (23). However, our study demonstrated the analysis of metabolites in plasma samples at an advanced stage of kidney disease, in which sample mixtures are more complex and challenging, as compared to the previous study. Therefore, the use of HR-GC × GC-TOFMS analysis could provide the opportunity to gain deeper metabolite information in other complicated diseases in comparison to the traditional GC technique.

Previous studies have explored the blood metabolite profiles of patients with predialysis CKD and DM using LC-MS/MS (15, 24) and GC-MS (14). To our knowledge, this study is the first to investigate plasma metabolites in DM patients with established KF, using the GC × GC-TOFMS method. Plasma metabolomic analysis of the DM with KF group revealed a different profile, whereas no difference was observed between the CON and DM groups. This may be due to the insignificant difference between the demographic data of the CON and DM groups in terms of kidney function parameters including creatinine, eGFR, albumin, and total protein (Table 1). In addition, four sugar alcohols (D-sorbitol, D-dulcitol or D-galactitol, and D-maltitol) were verified in the unique analysis, and 10 (of the 23) significant metabolites (Table 2) belonged to sugars and sugar alcohols, some of which have been previously reported to be linked to DM-related predialysis CKD progression (15, 16). Typically, chronic hyperglycemia can activate multiple collateral glucose-utilizing pathways, such as the polyol pathway, protein kinase C pathway, advanced glycation end products formation, hexosamine biosynthetic pathway, pentose phosphate pathway, and anaerobic glycolytic pathway. Activation of these aberrant pathways and associated oxidative stress generation are central to the pathogenesis of macrovascular and microvascular complications in DM (25, 26). In this study, we found significantly higher levels of sugars and sugar alcohols (D-galactose, sucrose, D-fructose, D-dulcitol, D-sorbitol, and myo-inositol) in the DM with KF group, as compared to those in the DM with normal kidney function and control groups. The findings of increased erythritol, arabitol, and mannitol levels were consistent with those of previous studies in dialysis patients (27). A more recent study using GC-MS also identified elevated levels of D-maltose, D-ribose, erythritol, myo-inositol, L-arabitol, mannitol, and L-arabinose in DM patients, as compared to those in moderate degree of DKD patients *versus* DM patients without kidney disease (14). The elevated levels of sugar and sugar alcohol metabolites may be linked to diet (especially erythritol, which could be used as an artificial sweeteners), altered metabolic pathways, decreased renal clearance, and gut dysbiosis. The latter is increasingly recognized as being altered in CKD and KF (28, 29). The accumulation of some of these sugar alcohols and sugars may contribute to the long-term complications of DM with KF. Blood glucose levels may vary rapidly with meals and still remain within the normal range. In the polyol pathway, hyperglycemia or excess glucose is converted to sorbitol by aldose reductase (30, 31). In the presence of sorbitol dehydrogenase, sorbitol is converted to fructose. However, the retina, nerves, and kidneys are tissues that have low sorbitol dehydrogenase levels, limiting the conversion of sorbitol to fructose. The accumulation of sorbitol in cells leads to the development of retinopathy, peripheral neuropathy, and nephropathy, respectively. Moreover, aldose reductase also converts galactose to dulcitol (galactitol) (32). Both elevated sorbitol and dulcitol have been linked to increased cataract formation. Another metabolite, mesoerythritol has recently been shown to be synthesized from glucose *via* the pentose phosphate pathway. Serum erythritol has been demonstrated to predictive DKD and coronary events in non-CKD cohorts; however, its role as a predictor of adverse outcome in KF is not yet known (33).

Although metabolite profiles and clinical parameters, such as eGFR and creatinine, could not be used to differentiate between the CON and DM groups, a distinct correlation pattern among those was observed in each group. This finding highlights the promise of integrative analysis of omics and clinical data. In our case, the small magnitude of the changes in metabolites and clinical characteristics precluded the separation between the CON and DM. Taking the relationships of molecular and clinical level data into consideration may pave the way for the description of disease status. Interestingly, although the total bilirubin levels were not distinguishable between the groups, its correlation pattern stood out in the DM with KF group. However, currently, there is no compelling evidence for a link between total bilirubin levels and CKD/KF progression outcomes.

In summary, we validated the GC × GC-TOFMS method and demonstrated its use for plasma metabolomics analysis in the DM with KF. We have identified several metabolites among sugars and sugar alcohols that were significantly elevated in the DM with KF group, as compared to those in the CON and DM groups, respectively. The galactose metabolism and polyol pathway were found to be the most induced metabolic pathways, with the accumulation of cellular sugar alcohols, potentially being an underlying mechanism of CKD progression to KF. The identification of sugar and sugar alcohol metabolites in this study provides crucial information that could potentially be useful for identification of future metabolite biomarkers for the early detection of DM to KF progression. However, further studies are needed to validate these potential biomarkers in earlier stages and in a larger cohort.

## Limitations of the study and future perspectives

This study has several limitations. First, the cohort had a small sample size and there were missing data for clinical parameters during follow-up. These parameters may affect the metabolomics analysis. Many pathological events can occur in the biological systems of the human body during the development of CKD leading to KF, such as microalbuminuria, macroalbuminuria, declining eGFR, and other different causes of CKD progression. Secondly, the lack of classification of sample groups in the early stages of CKD should be improved upon for the possible detection of early stage biomarkers. The first step toward proving that the candidate metabolites found in this study can be used as early stage biomarkers would be to perform a targeted quantitative analysis of these identified metabolites. Large-scale investigation of metabolites of interest in various patient groups should then be performed and validated on a second set of cohorts for further study.

## Experimental procedures

### Study design

This is a cross-sectional case-control study

### Subjects

All participants provided informed consent before participating in the study. This study was approved by the Ethical Clearance Committee on Human Rights Related to Research Involving Human Subjects, Faculty of Medicine, Ramathibodi Hospital, Mahidol University (COA. MURA2021/643). The human studies reported in this study abide by the Declaration of Helsinki principles. The study included three groups (N = 20 in each group) as follows: (I) CON, (II) DM (diabetes with normal kidney function), and (III) DM with KF (diabetes with kidney failure). The CON group comprised healthy male and female volunteers. All the subjects in the CON group had normal renal function (eGFR≥60 ml/min/1.73 m^2^ and normal urinalysis [dipstick protein=negative]), and no DM. A patient was considered to have DM if their fasting blood sugar levels >126 mg/dl or if they had been taking oral hypoglycemic drugs/insulin. The DM with normal kidney function group consisted of individuals with normal GFR (eGFR ≥ 60 ml/min/1.73 m^2^) and normoalbuminuria. The DM with KF group included patients known to undergo DM and only kidney replacement therapy for more than 3 months. Patients with a history of kidney transplantation were excluded from the study. Patients with comorbidities, such as hypertension, and those on medications for cardiovascular disease or cancer were excluded from the study.

## Methods

### Plasma collection and preparation

Fresh blood samples were collected from each subject and stored in ethylenediaminetetraacetic. After centrifugation at 3500 rpm for 10 min, 100 μl of the plasma was aliquoted into an Eppendorf tube and stored at –80 °C until analysis.

### Chemical standards and reagents

Hexane, methanol (MeOH), methoxyamine hydrochloride (MeOX), N-methyl-N-(trimethylsilyl)-trifluoroacetamide (MSTFA) + 1% chlorotrimethylsilane (TMCS), N-tert-butyldimethylsilyl-N-methyltrifluoroacetamide (MTBSTFA), and reference standards, including amino acids, sugars, sugar alcohols, and free fatty acids, were purchased from Sigma–Aldrich. A list of the reference standards is provided in Table S4. Stable isotope-labeled internal standard (IS) compounds, including DL-alanine-3,3,3-d_3_ and L-phenylalanine-1-C_13_, were purchased from Sigma–Aldrich and Cambridge Isotope Laboratories Inc, respectively. Pyridine was purchased from Tokyo Chemical Industry Inc MeOX (15 μg/μl in pyridine) and standard solutions (2 mM, in Milli-Q water or in hexane or in 0.1 mM HCl) were freshly prepared before analysis. The IS compounds were prepared in MeOH, at a concentration of 20 ng/μl.

### Sample extraction and derivatization

Sample preparation was modified from the study of Jiye *et al*. (17). In brief, the frozen plasma samples were thawed on ice at room temperature (RT) for 30 min. The samples were then vortexed for 5 s. Plasma (100 μl) was added to a 1.5 ml Eppendorf tube (on an ice bath) and mixed with 900 μl of precooled 90% aqueous MeOH containing 20 ng/μl of the IS compounds. The mixture was then shaken using a vortex mixer (Scientific Industries) for 2 min. The samples were kept at –20 °C for 1 h, following which they were centrifuged for 10 min at 19,600×*g*, 4 °C. After centrifugation, 200 μl of the supernatant containing 3.6 μg of the IS compounds was transferred into a new Eppendorf tube and evaporated to dryness at 65 °C (∼2 h), using a Centrivap concentrator (Labconco). The samples were stored at –20 °C until further analysis. The pooled sample (QC) was prepared by mixing with 200 μl of each supernatant (from the plasma extract). Subsequently, the samples were subjected to a methoximation reaction, by adding 30 μl of MeOX in pyridine (15 μg/μl) to each sample. The sample was then sonicated at 25 °C for 3 min and left at RT for 16 h. The mixture was then added to 50 μl of MSTFA with 1% TMCS and sonicated for 3 min at RT. The mixture was then incubated at 70 °C for 1 h and then cooled down at RT for 20 to 30 min. The derivatized samples were transferred into a GC vial for GC-TOFMS and GC × GC-TOFMS analyses.

### GC-TOFMS and GC × GC-TOFMS analyses

The derivatized samples were analyzed by GC-TOFMS and GC × GC-TOFMS (Pegasus 4D HRT, Leco Corp. Inc). QC samples were distributed across the sample sequence (every 15 samples). One microliter of each sample was injected into the split mode (1:20) at 250 ^°^C. The QC samples were used to monitor the reproducibility of the measurements. For GC × GC-TOFMS analysis, the first column was a nonpolar Rxi-5sil MS column (5% diphenyl-methyl polysiloxane and 95% dimethylpolysiloxane), with 30 m length, 0.25 mm internal diameter, and 0.25 μM film thickness (Restek), while the second column was a Rxi-17sil MS column (50% phenyl methyl polysiloxane and 50% dimethylpolysiloxane), with 1 m length, 0.25 mm internal diameter, and 0.25 μM film thickness (Restek). The temperature programming for the first GC column in the GC × GC-TOFMS analysis was set as follows: initial temperature at 50 °C (5 min), increased to 180 °C at 25 °C/min (1 min), increased to 220 °C at 10 °C/min (1 min), increased to 260 °C at 15 °C/min, and finally increased to 300 °C at 15 °C/min (4 min). The secondary offset was set at 10 °C above the primary oven temperature. For the GC × GC-TOFMS analysis, the modulator temperature was set to 15 °C. The second-dimension separation time was set to 4 s, and hot and cold pulse durations were 0.8 s and 1.20 s, respectively. For the GC-TOFMS analysis, the first GC condition was the same as that for GC × GC the analysis, whereas the second GC column was inactive. Helium was used as the carrier gas at a flow rate of 1 ml/min. Electron ionization was performed at 70 eV, with an ion source temperature of 250 °C. Mass spectral data were collected in scan mode, ranging from 40 to 1200 *m/z* at a rate of 20 spectra/s for GC-TOFMS and 200 spectra/s for GC × GC-TOFMS. The solvent delay was set to 500 s.

### Data preprocessing, processing, and metabolite identification

The major steps of metabolomics data handling, such as data preprocessing, data processing, and data analysis and interpretation, were performed based on a known protocol (34). Briefly, raw data from the GC-TOFMS and GC × GC-TOFMS analyses were preprocessed using ChromaTOF (version: 5.50, Leco Corp.). For the untargeted analysis, the peak identification was achieved by comparing the experimental mass spectra with the reference spectra contained in the NIST mass spectral library (NIST 2017). The parameters for the peak-finding method were as follows: similarity matching score ≥ 700, signal-to-noise ratio ratio ≥ 20, peak width = 0.06 σ, and minimum number to retain the spectrum of peaks > 3. For the targeted analysis, the analyte finding parameters were as follows: similarity matching score ≥ 700, signal-to-noise ratio ≥ 50, peak width = 0.06 σ, and minimum number to retain the spectrum of peaks > 5. The target analyte detection methods with reference metabolite standards, including 20 amino acids, 10 sugars, 9 sugar alcohols, and 8 free fatty acids, were based on their retention times and mass spectra libraries. For plasma sample analysis, the features were classified into four levels of metabolite identification, which were as follows: (1) confidently identified compounds, (2) putatively annotated compounds, (3) putatively characterized compound classes, and (4) uncharacterized or unknown compounds (21). Level 1 metabolite identification was performed by comparing at least two orthogonal properties (*i.e.*, mass spectra, retention time, etc.) of unidentified features with the reference standards, whereas level 2 was identified based on spectral similarity against the NIST library, with a similarity index of at least 700 (70% matching). Features with a library matched score of less than 700 were classified as unknown compounds and were excluded from this analysis. Subsequently, the identified compounds (both levels 1 and 2) were mapped against the human metabolome database to identify their subclasses. For quantitative analysis, the area under the curve (AUC) of the identified metabolites in the experimental blank was subtracted from that in the real samples. The signals from the artifacts of the derivatization reaction, solvent peak (*i.e.*, heptane), and chemical signals from column bleeding were removed (35). Missing values (at least 30%) of the metabolites from each group were replaced with imputed values using median values. The AUC of the metabolites mass was normalized according to the crosscontribution compensating for the multiple standards normalization method (36) with two IS compounds, including DL-alanine-3,3,3-d_3_ and L-phenylalanine-1-C_13,_ using R software (version 1.3.959). Data were log-2 transformed and scaled using the pareto method prior to statistical analysis.

### Statistical analysis

Comparison of the performance of the GC-MS and GC × GC-TOFMS analysis in terms of number of metabolites identified, different subclasses of the identified metabolites, and mass coverage of the detected metabolites in pooled plasma samples between the CON and DM with KF groups was performed using the *t* test. Multivariate analyses, including principal component analysis and orthogonal partial least squares discriminant analysis (OPLS-DA), were performed using SIMCA (version 16.0; Umetrics). The validation of the models was reported as R^2^ (goodness of fit) and Q^2^ (goodness of prediction), and the statistical significance of each model was estimated using a permutation test (n = 500) (37). Two OPLS-DA models were constructed for comparison: (1) DM and CON and (2) DM with KF and CON. The SUS plot was generated by combining the correlation loadings from both the OPLS-DA models. The plot represents contributions of the metabolites to DM with KF and DM, as compared to that of a common reference (or CON). The contributions were computed as *p*(corr) values and the metabolites were selected with respect to their position in the plot. The shared and unique metabolites corresponding to the study groups were identified from the SUS plot using *p*(corr) values (22). *p*(corr) is the scaled loadings (-1.0–1.0) that indicates the correlation coefficient between each variable and the model (38). Metabolites with an absolute *p*(corr) value > 0.7 that were then validated with reference standards were selected for pathway enrichment analysis. Differences in the normalized AUCs of the metabolites among pairwise comparisons and all-group comparisons were determined using the Mann–Whitney U-test and Kruskal–Wallis test, respectively. Spearman's correlation coefficient was used to analyze the correlations between metabolites and clinical parameters were analyzed using the Spearman’s correlation method implemented in R programming. MetaboAnalyst 5.0, which is an integrative platform for statistical and functional analysis of metabolomics data (39, 40, 41), was used to perform the enrichment analysis. Based on the Kyoto Encyclopedia of Genes and Genome (KEGG) analysis revealed the possibility of specific biological pathways related to the disease pathogenesis of the disease (42). A *p*-value <0.05 was used to determine significant pathways in the KEGG analysis.

## Data availability

All relevant data are contained within this research article and in the supporting information.

## Supporting information

This article contains supporting information.

## Conflict of interest

The authors declare that they have no conflicts of interest with the contents of this article.
