# Supplementary material for: GC × GC-TOFMS metabolomics analysis identifies elevated levels of plasma sugars and sugar alcohols in diabetic mellitus patients with kidney failure
Source: J Biol Chem. 2022 Aug 31;298(10):102445. doi: 10.1016/j.jbc.2022.102445 (PMC9531178; doi:10.1016/j.jbc.2022.102445)
Supplement: Supporting Information [file mmc1.docx]

**Supplementary information**

**GC×GC-TOFMS Metabolomics Analysis Identifies Elevated Levels of Plasma Sugars and Sugar Alcohols in Diabetic Mellitus Patients with Kidney Failure**

Kassaporn Duangkumpha^1,2^, Narumol Jariyasopit^1,2^, Kwanjeera Wanichthanarak^1,2^, Esha Dhakal^1,2^, Pattipong Wisanpitayakorn^1,2^, Sansanee Thotsiri^3^, Yongyut Sirivatanauksorn^2^, Chagriya Kitiyakara^4,6^, Nuankanya Sathirapongsasuti^5,6^, Sakda Khoomrung^1,2,7, *^

^1^Metabolomics and Systems Biology, Department of Biochemistry, Faculty of Medicine Siriraj Hospital, Mahidol University, Bangkok 10700, Thailand

^2^Siriraj Metabolomics and Phenomics Center, Faculty of Medicine Siriraj Hospital, Mahidol University, Bangkok 10700, Thailand

^3^Department of Medicine, Ramathibodi Hospital, Mahidol University, Bangkok, Thailand

^4^Section of Translational Medicine, Faculty of Medicine Ramathibodi Hospital, Mahidol University, Bangkok, Thailand

^5^Research Network of NANOTEC - MU Ramathibodi on Nanomedicine, Bangkok, Thailand

^6^Center of Excellence for Innovation in Chemistry (PERCH-CIC), Faculty of Science, Mahidol University, Bangkok, Thailand

**Running title:** GC×GC-TOFMS for plasma metabolomics analysis of DM with KF

**Keywords:** Metabolomics, GC×GC-TOFMS, kidney failure, diabetic mellitus (DM)


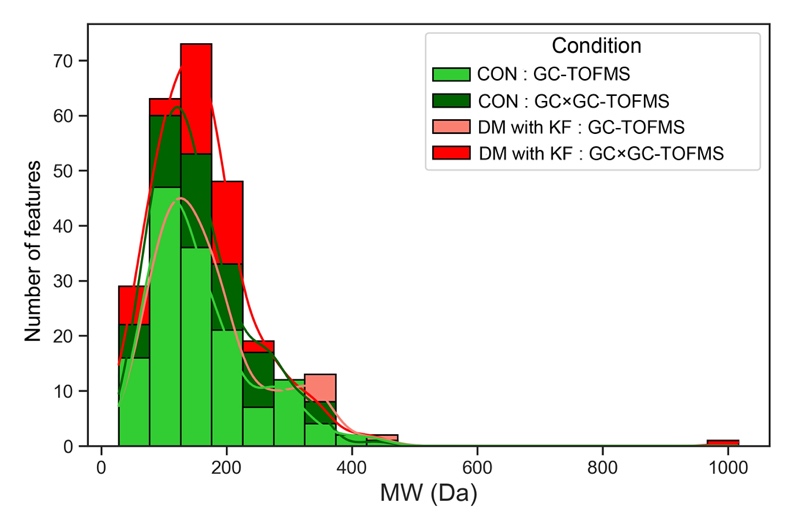


**Figure S1** The number of identified features in the range of molecular weight (Da) in pooled plasma samples in both CON and DM with KF groups compared between GC-TOFMS and GC×GC-TOFMS analysis.


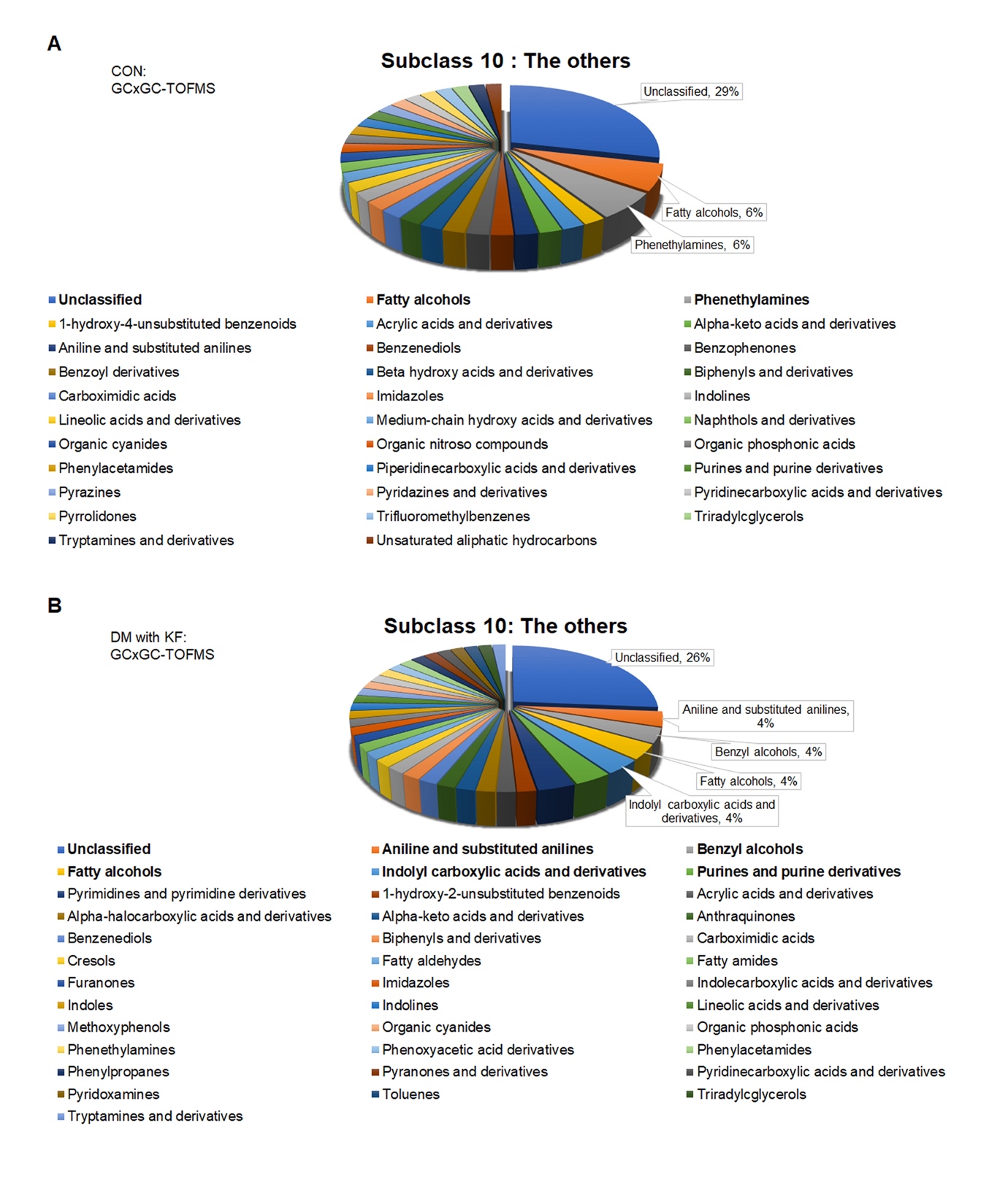


**Figure S2** Pie chart of the other subclasses of GC×GC-TOFMS analysis in both CON (A) and DM with KF groups (B)


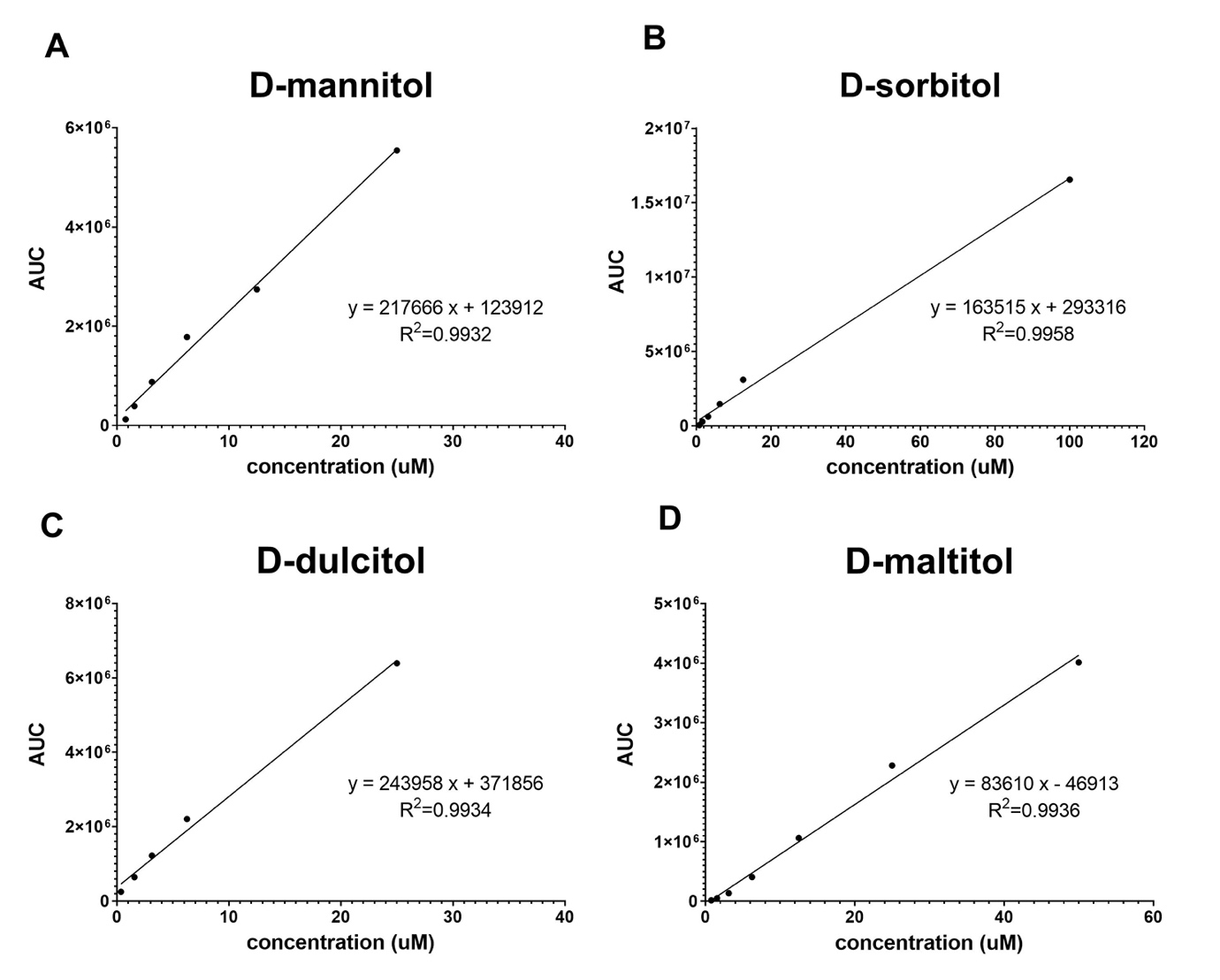


**Figure S3** The calibration curves of four unique including D-mannitol (A), D-sorbitol (B), D-dulcitol (C), and D-maltitol (D).


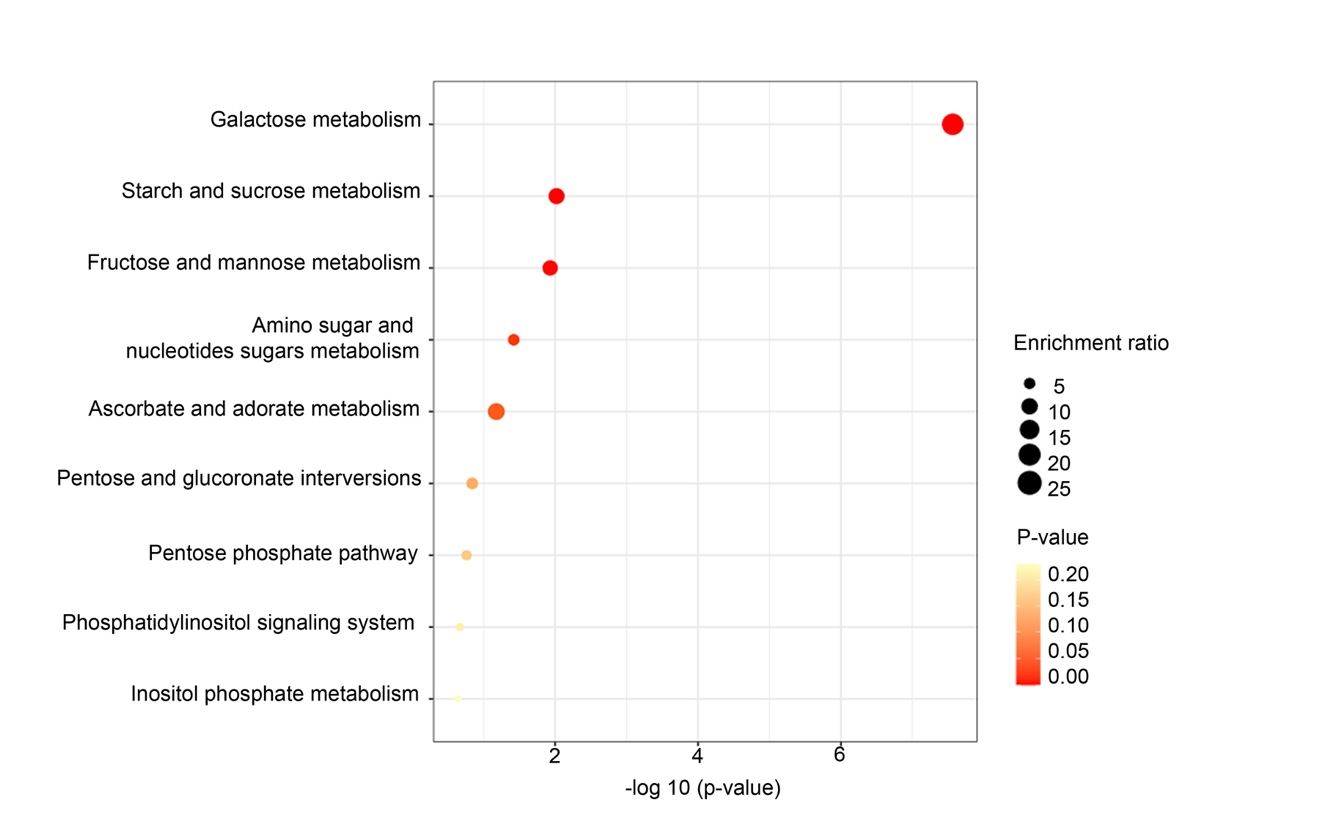


**Figure S4** Enrichment analysis of 14 potential metabolites of DM with KF patients based on KEGG database

**Supplementary table**

**Table S1** Number of metabolites identified in different subclasses in both CON and DM with KF groups in comparison between GC-TOFMS and GC×GC-TOFMS analysis. (excel file)

**Table S2** The normalized AUC of 89 metabolites identified from the two levels of metabolite identification. (excel file)

**Table S3** The enrichment analysis based on KEGG database of 14 potential metabolites related to DM with KF.

| **Pathways** | **Metabolite Set** | **Total** | **Hits** | **Expect** | **P value** | **FDR** | **REF** | **Detail** |
| --- | --- | --- | --- | --- | --- | --- | --- | --- |
| Galactose metabolism* | D-galactose, D-fructose, D-dulcitol, D-sorbitol, Myo-inositol | 27 | 6 | 0.231 | 0.000 | 0.000 | KEGG | Stachyose; D-Tagatose 6-phosphate; D-Gal alpha 1->6D-Gal alpha 1->6D-Glucose; Sucrose; Raffinose; Melibiose; D-Galactose; Galactosylglycerol; Epimelibiose; Melibiitol; alpha-D-Galactosyl-(1->3)-1D-myo-inositol; Alpha-D-Glucose; Alpha-Lactose; Glucose 1-phosphate; Uridine diphosphategalactose; Uridine diphosphate glucose; Galactose 1-phosphate; D-Galactose; Glucose 6-phosphate; D-Tagatose 1,6-bisphosphate; D-Glucose; D-Fructose; Galactitol; Glycerol; D-Mannose; Sorbitol; myo-Inositol |
| Starch and sucrose metabolism* | D-fructose, D-maltose | 18 | 2 | 0.154 | 0.010 | 0.329 | KEGG | Cellodextrin; Cellobiose; D-Fructose; Sucrose; beta-D-Glucoside; Uridine diphosphate glucose; Glucose 1-phosphate; Glucose 6-phosphate; D-Glucose; Amylose; Trehalose; Maltodextrin; Starch; D-Maltose; Dextrin; Isomaltose; Fructose 6-phosphate; Alpha-D-Glucose 1,6-bisphosphate |
| Fructose and mannose metabolism* | D-sorbitol, D-fructose | 20 | 2 | 0.171 | 0.012 | 0.329 | KEGG | **Sorbitol**; D-Fructose; D-Mannose; D-Fructose 2,6-bisphosphate; Mannose 6-phosphate; Fucose 1-phosphate; GDP-4-Dehydro-6-deoxy-D-mannose; Guanosine diphosphate mannose; D-Mannose 1-phosphate; Beta-D-Fructose 6-phosphate; D-Glyceraldehyde 3-phosphate; Fructose 1-phosphate; Glyceraldehyde; beta-D-Fructose 1,6-bisphosphate; L-Fucose; L-Fuconate; Alpha-D-Glucose; GDP-L-fucose; Dihydroxyacetone phosphate; 2-Dehydro-3-deoxy-L-fuconate |
| Amino sugar and nucleotide sugar metabolism* | D-Galactose, D-D-fructose | 37 | 2 | 0.316 | 0.038 | 0.796 | KEGG | N-Acetyl-D-glucosamine; N-Acetyl-D-Glucosamine 6-Phosphate; N-Acetyl-glucosamine 1-phosphate; UDP-N-acetyl-alpha-D-glucosamine; N-Acetylmannosamine; N-Acetylneuraminic acid 9-phosphate; N-Acetylneuraminic acid; Galactose 1-phosphate; Uridine diphosphate glucose; Glucose 1-phosphate; Uridine diphosphate glucuronic acid; Glucosamine 6-phosphate; Mannose 6-phosphate; Guanosine diphosphate mannose; Fructose 6-phosphate; Alpha-D-Glucose; D-Galactose; D-Mannose 1-phosphate; GDP-L-fucose; Fucose 1-phosphate; L-Fucose; N-Glycolylneuraminic acid; Ferricytochrome b5; Chitobiose; Chitin; Glucose 6-phosphate; D-Mannose; D-Fructose; Glucosamine; N-Acetyl-D-mannosamine 6-phosphate; Cytidine monophosphate N-acetylneuraminic acid; Uridine diphosphategalactose; UDP-D-Xylose; Beta-D-Fructose 6-phosphate; GDP-4-Dehydro-6-deoxy-D-mannose; CMP-N-glycoloylneuraminate; Ferrocytochrome b5 |
| Ascorbate and aldarate metabolism | Myo-inositol | 8 | 1 | 0.068 | 0.067 | 1.000 | KEGG | **myo-Inositol**; Uridine diphosphate glucose; L-Gulonolactone; D-Glucurono-6,3-lactone; Uridine diphosphate glucuronic acid; D-Glucuronic acid; Gulonic acid; Glucaric acid |
| Pentose and glucuronate interconversions | D-xylose | 18 | 1 | 0.154 | 0.144 | 1.000 | KEGG | beta-D-Glucuronoside; D-Ribulose 5-phosphate; D-Ribitol 5-phosphate; D-Xylulose; L-Arabitol; D-Xylitol; Gulonic acid; Glucose 1-phosphate; Uridine diphosphate glucuronic acid; Uridine diphosphate glucose; D-Xylose; D-Glucuronic acid; Xylulose 5-phosphate; CDP-ribitol; L-Arabinose; L-Threo-2-pentulose; 3-Dehydro-L-gulonate; D-Xylono-1,5-lactone |
| Pentose phosphate pathway | D-ribose | 22 | 1 | 0.188 | 0.173 | 1.000 | KEGG | Glucose 6-phosphate; Deoxyribose; Deoxyribose 1-phosphate; Deoxyribose 5-phosphate; D-Ribose 5-phosphate; Ribose 1-phosphate; D-Ribose; D-Sedoheptulose 7-phosphate; D-Glyceraldehyde 3-phosphate; D-Ribulose 5-phosphate; Beta-D-Fructose 6-phosphate; beta-D-Fructose 1,6-bisphosphate; 6-Phosphogluconic acid; 6-Phosphonoglucono-D-lactone; Beta-D-Glucose 6-phosphate; Gluconic acid; Gluconolactone; Glyceric acid; Phosphoribosyl pyrophosphate; Xylulose 5-phosphate; D-Erythrose 4-phosphate; 2-Phosphoglyceric acid |
| Phosphatidylinositol signaling system | Myo-inositol | 28 | 1 | 0.239 | 0.215 | 1.000 | KEGG | 1-Phosphatidyl-1D-myo-inositol 3-phosphate; Inositol 1,3,4,5,6-pentakisphosphate; 1-Phosphatidyl-D-myo-inositol; PIP2(16:0/16:0); myo-Inositol; CDP-diacylglycerol; 1-Phosphatidyl-1D-myo-inositol 4-phosphate; Phosphatidate; 1,2-Diacyl-sn-glycerol; 1-Phosphatidyl-1D-myo-inositol 3,4-bisphosphate; 1-Phosphatidyl-1D-myo-inositol 5-phosphate; 1D-Myo-inositol 1,3,4,6-tetrakisphosphate; 1D-Myo-inositol 1,3-bisphosphate; D-Myo-inositol 4-phosphate; Inositol 1-phosphate; 1D-myo-Inositol 3-phosphate; 1D-Myo-inositol 3,4-bisphosphate; 1D-Myo-inositol 1,4-bisphosphate; Inositol 1,4,5-trisphosphate; Inositol 1,3,4-trisphosphate; Inositol 1,3,4,5-tetraphosphate; Phosphatidylinositol-3,4,5-trisphosphate; 1-Phosphatidyl-1D-myo-inositol 3,5-bisphosphate; 1D-Myo-inositol 1,4,5,6-tetrakisphosphate; Myo-inositol hexakisphosphate; 1-Diphosinositol pentakisphosphate; 5-Diphosphoinositol pentakisphosphate; 1D-myo-Inositol 1,5-bis(diphosphate) 2,3,4,6-tetrakisphosphate |
| Inositol phosphate metabolism | Myo-inositol | 30 | 1 | 0.256 | 0.229 | 1.000 | KEGG | 1-Phosphatidyl-1D-myo-inositol 5-phosphate; PIP2(16:0/16:0); Inositol 1,4,5-trisphosphate; 1D-Myo-inositol 1,4,5,6-tetrakisphosphate; Phosphatidylinositol-3,4,5-trisphosphate; Inositol 1,3,4,5-tetraphosphate; Inositol 1,3,4-trisphosphate; 1D-Myo-inositol 1,3,4,6-tetrakisphosphate; D-Myo-inositol 3,4,5,6-tetrakisphosphate; 1D-Myo-inositol 1,4-bisphosphate; 1D-Myo-inositol 3,4-bisphosphate; 1D-Myo-inositol 1,3-bisphosphate; Glucose 6-phosphate; D-Myo-inositol 4-phosphate; 1D-myo-Inositol 3-phosphate; myo-Inositol; 1-Phosphatidyl-D-myo-inositol; 1-Phosphatidyl-1D-myo-inositol 4-phosphate; 1-Phosphatidyl-1D-myo-inositol 3-phosphate; Inositol 1-phosphate; Inositol 1,3,4,5,6-pentakisphosphate; Malonic semialdehyde; Dihydroxyacetone phosphate; 1-Phosphatidyl-1D-myo-inositol 3,4-bisphosphate; 1-Phosphatidyl-1D-myo-inositol 3,5-bisphosphate; D-Glucuronic acid; 1,2-Diacyl-sn-glycerol; Myo-inositol hexakisphosphate; Acetyl-CoA; D-Glyceraldehyde 3-phosphate |

*The significant related pathways with p<0.05

**Table S4** Chemical standards and reagents

| **Type of chemical** | **Name** | **Company** |
| --- | --- | --- |
| Derivatizing agents and solvents | Hexane,  Methanol (MeOH),  Methoxyamine hydrochloride (MeOX),  N-methyl-N-(trimethylsilyl)-Trifluoroacetamide (MSTFA) + 1% Chlorotrimethylsilane (TMCS)  Pyridine | Sigma-Aldrich (St. Louis, MO, USA)  Sigma-Aldrich (St. Louis, MO, USA)  Tokyo Chemical Industry, Inc (Tokyo, Japan) |
| Amino acid standards (20) | L-alanine, L-valine, L-leucine, L-isoleucine, L-tryptophan, L-tyrosine, L-glycine, L-glutamine, L-glutamic acid, L-methionine, L-arginine, L-cystine, L-lysine, L-proline, L-threonine, L-serine, L-asparagine, L-phenylalanine, L-aspartic acid, L-histidine | Sigma-Aldrich (St. Louis, MO, USA) |
| Free fatty acids (8) | myristic acid, palmitic acid, palmitelaidic acid, oleic acid, linoleic acid, α-linolenic acid, stearic acid, arachidonic acid | Sigma-Aldrich (St. Louis, MO, USA) |
| Sugars (10) | D-glucose, D-galactose, D-tagatose, D-talose, D-mannose, D-xylose, D-arabinose, D-ribose, D-fructose, D-maltose | Sigma-Aldrich (St. Louis, MO, USA) |
| Sugar alcohols (9) | D-arabitol, D-maltitol, D-mannitol, D-xylitol, D-adonitol, Meso-erythritol, Myo-inositol, D-sorbitol, D-dulcitol | Sigma-Aldrich (St. Louis, MO, USA) |
| The internal standard (IS) | DL-alanine-3,3,3-d_3_  L-phenylalanine-1-C_13_ | Sigma-Aldrich (St. Louis, MO, USA)  Cambridge Isotope Laboratories, Inc. (Andover, MA, USA) |
